# Connexins and Aging-Associated Respiratory Disorders: The Role in Intercellular Communications

**DOI:** 10.3390/biomedicines12112599

**Published:** 2024-11-13

**Authors:** Tatiana Zubareva, Ekaterina Mironova, Anna Panfilova, Yulia Krylova, Gianluigi Mazzoccoli, Maria Greta Pia Marasco, Igor Kvetnoy, Peter Yablonsky

**Affiliations:** 1 Department of Translational Biomedicine, Saint-Petersburg Research Institute of Phthisiopulmonology, 191036 Saint Petersburg, Russia; 2 Department of Biogerontology, Saint Petersburg Institute of Bioregulation and Gerontology, 197110 Saint Petersburg, Russia; 3 Medical Institute, Saint-Petersburg State University, 199034 Saint Petersburg, Russia; 4 Department of Pathology, Pavlov First St. Petersburg State Medical University, 197022 Saint Petersburg, Russia; 5 Chronobiologi Laboratory, Fondazione IRCCS Casa Sollievo della Sofferenza, Viale Cappuccini, 71013 San Giovanni Rotondo, Italy

**Keywords:** connexin, Cx43, Cx37, connexon, gap junction, intercellular communications, signaling molecules, respiratory system diseases, target therapy

## Abstract

This article reviews the contemporary understanding of the functional role of connexins in intercellular communications, their involvement in maintaining cellular and tissue homeostasis, and in aging-associated respiratory disease pathogenesis. Connexins are discussed as potential therapeutic targets. The review particularly focuses on the involvement of gap junction connexins and hemichannels in the transfer of calcium ions, metabolite molecules, ATP, and mitochondria through the cell membrane. Various disorders in the regulation of intercellular communication can heavily contribute to the pathogenesis of multiple diseases, including respiratory system diseases. A deeper understanding of molecular mechanisms underlying the activities of various connexins in gap junction channels will enable the prospective development of therapeutic approaches by either inhibiting or stimulating the activities of a certain connexin, while considering its critical functions in intercellular communications on the whole.

## 1. Introduction

Connexins are a family of gap junction proteins, represented in humans by molecule type 21. Connexins have a similar structure, consisting of one N-cytoplasmic terminus, two extra-cellular loops, one cytoplasmic loop, four transmembrane domains, and one C-terminal tail [1]. Amino acid sequences EL1 and EL2 (extracellular loops 1 and 2) are similar in different types of connexins, while the sequences of C-terminal tails of different connexin isotypes may vary [2]. Gap junctions (GJ) are the simplest in their structure among all types of intercellular communication; they perform multiple functions and participate in the specific activity of most cells, both in a normal physiological state and under pathological conditions. Since 2007, the GJ nomenclature system has been used for the genes that encode connexins, while the nomenclature of connexin proteins preserved their naming according to protein molecular weights. Thus, Cx43 is the connexin protein of 43 kDa. The gene encoding this connexin has the nomenclature name *GJA1* (Gap Junction protein, Alpha1), which is based on the group of α (GJA) and β (GJB) forms followed by an ID number.

**Figure 1:**
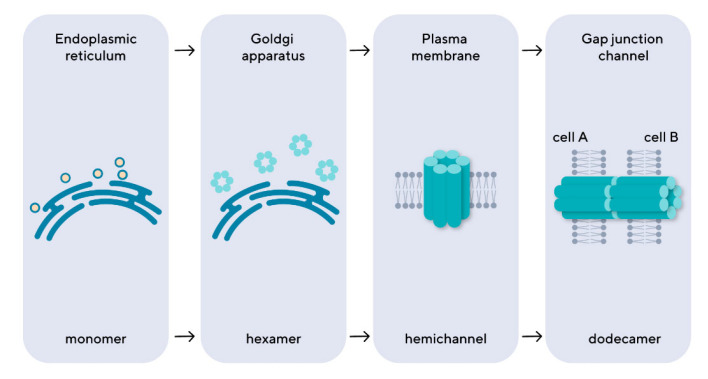
The intracellular stages of gap junction formation. The connexins are synthesized as monomers in the endoplasmic reticulum, then transported to the Goldgi apparatus, where they assemble into hexameric structures, connexons, which are then exported to the cell membrane, forming a hemichannel. The connexons of the neighboring cells form an intercellular channel of gap junctions by connecting with each other.

Connexins are assembled in the endoplasmic reticulum or Goldgi apparatus into hexametric arrays called connexons, which are then transported to the cell surface (Figure 1) [1]. Gap junctions are protein channels of 16–20 angstroms in diameter, which are formed by connexons, or hemichannels, of two communicating cells. Each connexon consists of six protein subunits, or connexins. It is presumed that the extracellular loops of opposite connexins bind to each other via antiparallel β sheet, thereby forming a β-barrel.

**Figure 2:**
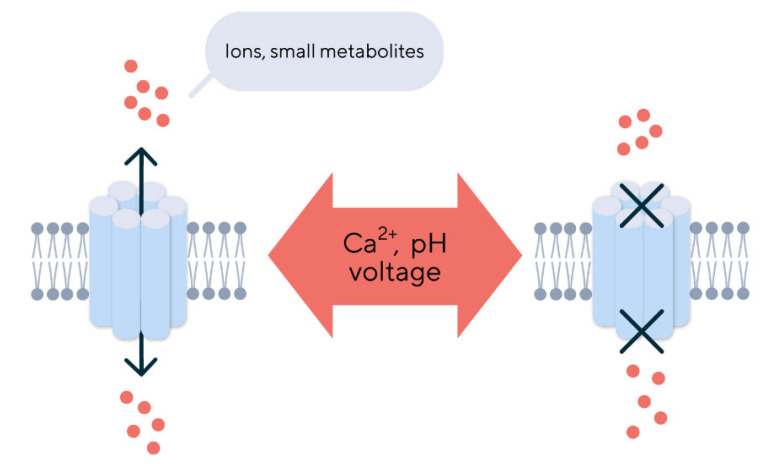
The regulation of gap junction operation. The opening and closing of gap junctions are regulated by extracellular and intracellular factors such as pH, calcium ion concentration, and connexin phosphorylation. Gap junctions serve to move ions and small molecules up to 1.2 kDa between adjacent cells. The cells can exchange molecules through gap junctions such as carbohydrates, nucleotides, second messengers (cAMP or cGMP), small peptides, and RNA.

Gap junctions enable communication between cells to exchange small metabolites and play an important role in regulating proliferation, cell differentiation, and maintaining tissue homeostasis. They also ensure the ions and small molecules exchange between the intracellular and extracellular medium [3,4]. The channel operation mechanism relies on a similar principle for connexins. The regulation of the open/closed state depends on the interaction between its cytoplasmic loop and cytoplasmic tail [5]. Interactions between the last nine amino acids at the end of the protein cytoplasmic tail and the L2 domain, located on the cytoplasmic loop, determine the opening of the hemichannel [6]. The operation of this molecular mechanism also depends on the intracellular concentration of calcium, changes in the voltage of the cell membrane, and the pH inside the cell (Figure 2) [7].

The gap junction regulation is performed by several extracellular and intracellular factors [8,9]. In the case of rapid regulation, the channel state is changed according to the open/closed principle. In the case of slow regulation, changes occur in the composition of connexin structures on membrane cells, the rate of post-translational modifications, and the rate of structural degradation.

One of the most significant intracellular regulation factors is the pH level. Thus, most gap junctions remain inactivated in the pH range of 7.3 to 6.49. An important exception here is connexons with Cx36 prevalence being blocked in the alkaline extracellular medium with pH > 7.5, which may explain their open state during ischemia [10]. Another important regulator of gap junction activity is phosphorylation of cytoplasmic domains of the connexon, which leads to channel blocking.

Connexin phosphorylation at several serine residues can induce conformational changes, thereby reducing the permeability of the gap junction for small organic molecules [11].

A connexon may consist of connexins of one type (a homotypic variant) or different connexins (a heterotypic variant). A gap junction formed by homotypic connexons is defined as homomeric; different connexons form a heteromeric channel. This peculiarity determines a range of cells between which intercellular communication is possible [12].

Cryo-electron microscopy (cryo-EM) studies have revealed four distinct conformations of Cx43, which determine dynamic changes in the size and surface properties of the gap duct. Such details not only suggest that channel activity can be regulated by conformational changes in individual protomers, but also explain the potential role of cholesterols in channel regulation, the formation of additional pores in the membrane, the structure of interconnexon docking interactions, and the evolutionary conservation of structural changes [13].

Detailed information on the structural configuration of GJ and HC is critical for understanding channel function and assessing the pathological nature of mutations in connexin genes associated with diseases and the development of new therapeutic methods involving connexins [14].

## 2. Cellular Localization of Connexins

**Figure 3:**
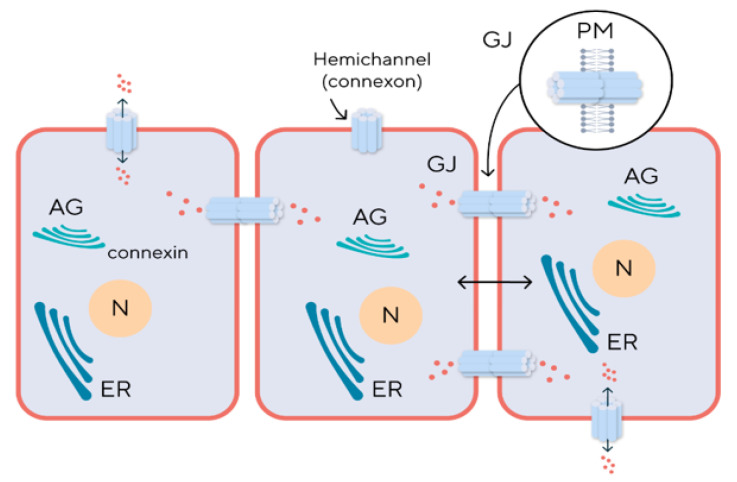
Intracellular localization and intercellular functioning of connexins. Abbreviation: AG—Apparatus of Goldgi, N—Nucleus, ER—Endoplasmic Reticulum, GJ—Gap Junction Channel, PM—Plasma Membrane.

Cytoplasmic and membrane expression of connexins has been determined for most cell types, some residing in the nucleoplasm (Figure 3). For example, Cx43 is formed in the Goldgi apparatus, which enables us to create a model for studying the role of this protein with brefeldin A, a Goldgi apparatus disruptor. Cx26 (connexin 26) is modified in the endoplasmic reticulum, which deprives it of specific inhibitors, and therefore its experimental study requires laboratory animals knocked out for the *GJB2* gene that encodes Cx26 [15].

Connexins have a relatively short half-life of only a few hours. It has been suggested that such a dynamic cycle of connexin synthesis and substitution allows for more finely regulated physiological processes to take place in cells [4]. Post-translational modification of connexins can occur in different ways which depend on the molecular weight of each protein. It has been demonstrated that Cx43 is modified by SUMO, which is presumed to stabilize Cx43 and cause the gap channel size to increase.

With age, there are a number of changes in the function and expression of connexins, which may affect the development of age-related pathologies. This is due to the fact that aging is accompanied by the accumulation of cellular stress, DNA damage, metabolic changes, and other factors that can affect the stability and functionality of connexin-dependent intercellular junctions.

One of the key aspects of changes in connexin function in the aging organism is the disruption of signaling. For example, it has been shown that with age, the expression of some types of connexins, such as Cx43, decreases, which can lead to deterioration in electrophysiological characteristics of tissues, such as myocardial conduction, which, in turn, increases the risk of arrhythmias and other cardiovascular diseases.

In addition, age-related changes in connexins can contribute to the development of neurodegenerative diseases. Some studies have found that the pathological regulation of connexins may be associated with the deterioration of cognitive function and an increased risk of diseases such as Alzheimer’s disease. Changes in the expression and function of connexins can disrupt the homeostasis of the neural network and worsen neuronal inflammatory processes.

## 3. Connexon Functions

Connexons (hemichannels) function outside the gap junction as well. The intracellular flow of Ca^2+^ goes through them; glutamate, ATP, and NAD^+^ are released into the extracellular space; large membrane pores are formed which are capable of passing ions and molecules through; and ATP-dependent release of Ca^2+^ occurs.

Hemichannel regulation is based on the same principles as in the gap junction, i.e., it is opened when the concentration of extracellular calcium decreases and the pH of the intercellular medium increases, along with an increased concentration of free radicals. Even though the range of substances transported by hemichannels is similar to that of gap junctions, they are supposed to be more prevalent and involved under pathological rather than physiological conditions [16].

Thus, it has been shown that the release of glutamate from astrocytes occurs mostly through hemichannels. And a decrease in the extracellular calcium concentration during ischemia is a signal for the opening of Cx43 hemichannels and the release of glutamate, NAD, and ATP from the cell, which, in turn, by coupling with P2X7 astrocyte receptors, enhances the release of a toxic neurotransmitter from cells several times [17].

Purinergic signaling is now considered to be a key factor in the inflammatory process. The mechanisms mediating the release of ATP into the extracellular space from cells of damaged or infected tissue sites are involved in the further activation of immunocompetent cells and the regulation of the immune response, as well as in the process of fibrosis and pain syndrome associated with chronic inflammation. Therefore, the specific modulation of ATP release mechanisms can be a foundation for a therapeutic treatment model of various inflammatory diseases [18].

## 4. Connexins and Aging

Recent studies have shown that the expression of connexins, primarily CX43, decreases in humans and animals during natural aging. The expression of connexins Cx32 and Cx26 in cardiomyocytes and hepatocytes of experimental animals also tended to decrease with aging without the involvement of genetic mutations or age-related diseases. Moreover, the decrease in the amount of connexin protein itself did not correlate with the amount of mRNA, which remained at the same level [19].

One of the possible reasons for this trend is the physiological adaptation of cells, where the reduction in the expression of connexins in the cells of the liver and heart induces a decrease in intercellular communication. And as a result, the possibility of distribution of senescence-associated secretory phenotype molecules also decreases, which can be regarded as a geroprotective mechanism. Also, a possible reason for the reduction in intercellular connexin-mediated channels is the need to reduce the cell’s loss of important functional molecules, such as ATP, glucose, or ions. And if the liver gap junctions are crucial for the synthesis of plasma proteins and the biotransformation of xenobiotics, then the myocardium in these channels coordinate the depolarization of cardiac cells, allowing them to work as a syncytium.

Immunohistochemical studies revealed a uniform distribution of Cx43 predominantly on the lateral sides of the atrial cardiomyocytes of young guinea pigs. In contrast, uneven distribution and mislocalization, as well as decreased Cx43 protein expression, were found in the atria of aged animals. These analyses suggest that age-related declines in atrial Cx43 and increases in extracellular matrix metalloproteinase-2 (MMP-2), as well as the disruption of Cx43, may contribute to the development of atrial fibrillation, the most common cardiac arrhythmia, especially in older adults [20].

Studies using experimental models have revealed significant sex differences in the amount of Cx43 protein in cardiomyocytes in groups of aging male and female rats. Cx43 expression was significantly lower in males compared to females, which correlated with conduction defects and myocardial contractile dysfunction due to the disruption of intercellular electrical and metabolic interactions involving connexins [21].

A decrease in the expression of connexin 43 can be considered as an integral indicator of aging, since this trend is observed in various tissues of the body. Thus, the expression of this protein was reduced by approximately 10 times in the teeth of elderly patients, which may be associated with the loss of vitality of the dental pulp with age and can be regarded as one of the characteristics of the aging pulp [22].

Reduced expression of Cx43 as a biomarker of cellular aging is also confirmed in studies with HEL-299 fibroblast cell cultures. A decrease in the amount of protein in aging fibroblasts during replicative aging was determined by Western blot in cell extracts and indirect fluorescence in cell culture [23].

Neuronal damage in neurodegenerative diseases may also be associated with abnormal changes in connexins in glia, resulting in glia losing their ability to support and protect neurons, and the resulting abnormal increase in ion and metabolite levels (Ca^2+^, glutamate, and ATP) ultimately leads to neuronal death [24].

To understand the role of connexins in the aging process, it should be considered that different connexins, localized at different cellular levels and in different tissues and differently organized at gap junctions, can participate in different pathways regulating intercellular interactions. This makes it important to determine the involvement of a specific connexin in these mechanisms, which could help prevent early aging or become the basis for treating diseases associated with aging.

## 5. The Expression and Role of Connexins in Respiratory Pathology

As we know, connexin 43 is involved in the development of organs such as the brain, heart, kidney, and lung [25,26]. Cx43 is expressed in mouse embryos already on the 14th day in the respiratory tract epithelium, type I and II alveolar cells, lung endothelium, lung fibroblasts, and lung smooth muscle cells [27]. Alveolar stenosis and late alveolar development have been observed in Cx43-deficient mice due to reduced expression of surfactant-associated protein C and smooth muscle alpha-actin [28]. Gap junctions are crucial for the intercellular propagation of calcium signals to regulate lung functions such as cilia movement and surfactant secretion [27]. In the absence of Cx43 expression, calcium transfer between alveoli and lung capillaries is altered [29]. Cx43 may play a role in lung remodeling, as evidenced by an increased expression of Cx43 by alveolar epithelial cells after radiation-induced pulmonary fibrosis [30].

Given the proven importance of Cx43 in the development of the respiratory system, particularly the lung alveoli, special attention should be paid to this connexin’s role in the pathogenesis of various forms of respiratory insufficiency leading to impaired gas exchange, hypoxia, and, consequently, further metabolic disorders in cells and tissues.

Acute respiratory distress syndrome (ARDS) is a lung disease whose definition is based on the degree of hypoxemia and pulmonary function tests [31]. In ARDS, the lung function is impaired due to an injury of the alveolar epithelium and capillary endothelium [32]. Respiratory insufficiency found in ARDS may occur due to the impairment of arterial oxygenation caused by damage of the alveolar-capillary barrier and an increased permeability of respiratory endothelial and epithelial cells, which results in neutrophil infiltration [33]. The gap junction proteins, especially Cx43, play an important role for the correct work of endothelial and epithelial cellular barriers in the lungs. The mechanism of this process is obviously much more complicated than just participation in localizing tight junctions and/or protein adhesion [34,35], as gap junctions modulate the vascular endothelium protective function indirectly through heterotypic interactions and with the help of ATP released through hemichannels [36]. Dephosphorylation of Cx43 in neutrophils can open Cx43 hemichannels and release ATP, which is one of the most significant inflammation factors, and promotes the strengthening of the barrier function in endothelial cells. The level of Cx43 expression affects the endothelial barrier function. Thus, if Cx43 expression decreases in vascular endothelial cells, then vascular barrier permeability decreases, and an increased expression of Cx43 leads to increased permeability of the barrier [37,38,39]. It has been demonstrated in a mouse model that neutrophils with open Cx43 channels have a higher level of recruitment, while inhibiting Cx43 could reduce neutrophil recruitment to the lungs [40]. In addition to affecting vascular permeability, Cx43 propagates anti-inflammatory signals by transmitting Ca^2+^ ions through hemichannels [12]. These findings enable us to consider Cx43 as a potential therapeutic target for restoring the respiratory function by preventing the neutrophil inflow.

Chronic obstructive pulmonary disease (COPD) is a common lung disease characterized by persistent respiratory symptoms which develop as a result of the impact of harmful particles and gasses. The only intercellular junctions that are injured in COPD are tight junctions. But given their close functional connection with gap junctions and the involvement of common auxiliary proteins, connexins play an important role in pathogenesis. The most common risk factor associated with COPD is the impact of cigarette smoke [41]. Inhalation of combustion particles will lead to an irreversible decrease in the proliferative capacity of cells, accompanied by the accumulation of p21 and p16 proteins that block the cell cycle, as well as a decrease in the expression of Cx43 and SIRT1, which dramatically reduces intercellular communication and contributes to epithelial aging and age-related respiratory diseases [42]. Connexins participate in the development of COPD by affecting intercellular communication in the endothelium. Thus, Cx43 and Cx37 expression decreases in response to nicotine exposure in human endothelial cells [43] and does not change in epithelial cell [44].

Cx37 connexin is an active participant in atherosclerotic processes associated with arterial disorders. It disappears from the endothelium of atherosclerotic plaques but can be found in macrophages recruited from lesions—that is, the expression of Cx37 changes in both mice and humans with atherosclerotic vascular disease. Cx37 hemichannel activity in primary monocytes and macrophages inhibits the adhesion of leukocytes by releasing ATP into the extracellular space. In other words, Cx37 hemichannels may control the initiation of the development of atherosclerotic plaques by regulating monocyte adhesion [45]. It has been found that connexin can improve the activity of macrophages by regulating the expression of chemokines in atherosclerotic mice, as well as increase the number of macrophages in inflammatory tissues and expand the area of plaque formation [46].

Connexins also play a significant role in asthma pathogenesis, chronic airway inflammation, followed by obstruction, shortness of breath, and labored breathing [47]. Allergic asthma includes sensitization to specific antigens (allergens) whose impact promotes the production of allergen-specific antibodies (IgE) which are bound with antigen, initiating an inflammatory cascade that leads to mast cell degranulation, eosinophil inflow, and cytokine release [48]. In a model of ovalbumin-induce allergic asthma, a sharp increase in Cx43 mRNA and the protein’s level was observed in asthmatic mice. Cx43 was mostly localized in the alveolar and bronchial epithelial layers [49]. Inhibiting Cx43 reduced the hyperactivity of airways as well as some specific markers of allergic asthma, such as eosinophil infiltration, Th2-cytokine levels, and IgE levels to ovalbumin in the blood serum [50]. A negative correlation was found between Cx37 expression in the lungs of asthmatic mice, including airway inflammation, airway responsiveness, and levels of Th2 cytokines in lungs [51]. The contribution of resident immunocompetent cells or cells recruited from the bloodstream to the initiation of asthmatic inflammation, as Cx43 is also actively expressed by resident lung cells such as epithelial, alveolar cells, and fibroblasts [52].

The expression of Cx43 as a signaling molecule is also important for the function of epithelial cells and alveolar macrophages which constitute the first line of defense from inhaled pathogens. Alveolar macrophages attached to the alveoli establish intercommunication through Ca^2+^ waves, using the epithelium as the conducting pathway [53]. The knockout of Cx43 enhances secretion of proinflammatory cytokines and, consequently, alveolar neutrophil recruitment.

## 6. Macrophage Connexins

Macrophages constitute the first line of defense against Mycobacterium tub. invasion in pulmonary tuberculosis. Upon encountering mycobacteria, they trigger their own apoptosis thereby releasing proinflammatory factors. Cx43 and Cx37 are the main connexins in macrophages that participate in the formation of gap junction channels which provide intercellular communication required for transmitting various cellular signals and functions during inflammation, and in the regulation of inflammatory cytokine synthesis and apoptosis [54]. Cx43 affects the mobility of macrophages—an increased expression of this protein enhances their migration ability [55]. Transmigration is associated with the formation of heterocellular gap junctions between macrophages and endothelial cells. A profound reduction in monocyte/macrophage transmigration across a blood–brain barrier model was observed when blocking Cx43 residing on the surfaces of those cells [56].

## 7. The Role of Connexins in Oncogenesis

The GJIC play a peculiarity contradictory role in the regulation of the tumor growth because they can act as tumor suppressor or pro-tumorigenic factors [57] and their different activity depends by their abundance, localization, tissue type, and which isoform of connexin is involved [58]. Different data defined the connexin tumor suppressor, in fact the deficiency of these molecules was underlined in mice with different types of tumors. So, the reduction in levels induces the malignant transformation of the cells acting on the alteration of the metabolism and inflammation and contribute to the development of the tumor [59]. Other types of connexin, when they lose their conformation or when they are upregulated, act as pro-tumorigenic and pro-metastatic factors [58].

Cx43 can act as an inhibitor at an earlier stage of lung cancer [60]. As we know, the expression of Cx43 mRNA and protein gradually decreases in the normal lung tissue adjacent to the tumor tissue—the Cx43 expression is usually lower in areas located closer to the tumor [61]. Thus, at an early stage, tumor cells have a negative impact on Cx43 expression in the surrounding normal lung cells. The primary tumor cells presumably “shut themselves out,” preventing intercellular communication with normal lung cells across gap junctions. However, in advanced carcinogenesis, Cx43 loses its function of a tumor suppressor gene and can be viewed as a conditional tumor suppressor [62]. As the expression rates of Cx43 decrease, the degree of cancer cells differentiation decreases, which enables them to easily propagate and metastasize, significantly worsening the disease progression prognosis [63]. Cx43 is considered to be a prognostic factor for progressing non-small cell lung cancer (NSCLC), as higher expression rates of Cx43 correlate with a favorable prognosis, while lower rates correlate with a negative prognosis [62].

Furthermore, connexins are involved in the regulation of endothelial cell stiffness, which counts for metastasis of lung cancer. Cx43 has been reported to be involved in this mechanism: upon the chemical inhibition of Cx43 functions, umbilical vein cells were activated [64], while an enhanced diapedesis of adenocarcinoma cells was observed. It means that Cx43 participates in decreasing the endothelial barrier function and facilitating diapedesis of tumor cells.

Connexin 37 is also involved in carcinogenesis, influencing the proliferation activity of tumor cells. Thus, unlike Cx43, Cx37 suppresses the proliferation of rat insulinoma cells, increasing the time required for each phase of the cell cycle. This happens only when connexin takes a specific conformation achieved by the interaction of the C terminus with a Cx37 pore-forming domain [65].

## 8. The Role of Connexins in Fibrosis Processes

The accumulation of fibrous connective tissue, as extracellular matrix components, in organs induces fibrosis, which can cause the loss of function of the organs [66]. This condition is characterized by the epithelial–mesenchymal transition, a process where the cells change by an epithelium to a mesenchymal phenotype. This change also affects some characteristics of the cells, in fact they became more prone to migration, invasion, and resistance to apoptosis [67]. The GJ and hemichannels are also involved in tissue homeostasis and restore homeostasis after damage, and some connexin can act independently by channels, influencing tissue regeneration and organ fibrosis [68].

Most respiratory diseases are accompanied by fibrosis and sclerosis processes. Cx43 is one of the most highly expressed connexins in fibroblasts. C43 is upregulated in alveoli during the acute phase of lung injury and in radiation-induced lung fibrosis [69]. Yet, it remains unclear whether this upregulation released with an increased level of Cx43 mRNA or with an increased number of cells [70]. However, Cx43 was significantly reduced in patients with idiopathic pulmonary fibrosis, while the mRNA level remained unchanged [71]. Cx43 is also involved in the proliferation and migration of pulmonary arterial fibroblasts in response to hypoxia [72].

Lung fibrosis in most cases is associated with inflammation which is characterized by an increased population of macrophages [73,74]. Macrophages secrete ATP, which, in turn, activates membrane channels for calcium ions inflow during inflammation [75]. It has been reported that Cx43 knockout in macrophages reduces the fibroblast response to cytosolic calcium in the joint cultivation of both types of cells [76], and that calcium wave propagation is significantly reduced in Cx43-deficient mice, Cx43 being one of the major connexins expressed by the pulmonary vasculature [27]. Cx37, in turn, is expressed in smooth muscle tracheal cells where it is colocalized with Cx43 but not expressed in fibroblast cells [77].

## 9. The Role of Connexins in Inflammatory Processes

In inflammation, destruction of the cell membrane leads to the release of many nucleotides because of their high intracellular content as compared to the extracellular space [78,79]. The release of ATP by necrotic cells creates a proinflammatory microenvironment by secreting proinflammatory cytokines and recruiting neutrophils to the site of necrosis [80]. Cx43 and Cx37 mediate the release of ATP into the extracellular space [81]. In addition, Cx43 spreads proinflammatory signaling by transporting Ca^2+^ between cells via hemichannels [82]. An increased level of endothelial Ca^2+^ induced in the alveolar capillary causes a secondary increase in Ca^2+^ in the adjacent capillary network and the adjacent venule, which is inhibited in the presence of peptides blocking Cx43 [29]. A possible proinflammatory role of these mechanisms is to activate the expression of venular P-selectin, an inflammatory adhesion molecule, which enables leukocyte recruitment to the endothelium [83] through Ca^2+^ diffusion between cells. Furthermore, increased thrombin-induced permeability of lung microvascular vessels in inflammation is completely suppressed by peptides that block Cx43, which also points to its important role in the inflammatory process [29].

Connexin 37 regulates monocyte adhesion, preventing the development of atherosclerotic lesions. The expression of Cx37 is altered in mouse and human atherosclerotic lesions; it is increased in macrophages recruited to the lesions and disappears from the endothelium of plaques. Cx37 deficiency alters profiles of differentially expressed genes in young mice toward the proinflammatory phenotype, which are then further affected in progressing atherosclerosis [84].

## 10. The Functional Relation Between Connexins and Mitochondria

It is known that connexins in general and Cx43 in particular play an important role in ARDS, COPD, and asthma, as described above. The mechanism of their action relies on calcium propagation and, possibly, other signaling from cell to cell across gap junctions, and on ATP release through the opening of hemichannels. It should be noted that, besides small molecules, antigen peptides [85] and microRNA [86] may be transferred between cells via gap junctions, which can also regulate multiple intracellular processes. Furthermore, as shown in a mouse model, Cx43 is involved in the formation of nanotubes through which mitochondria can be transferred from marrow stromal cells to alveolar cells [87,88]. Mitochondria play a crucial role in pathological processes as they are responsible for ATP formation and controlling reactive oxygen species. The Cx43 influences mitochondrial respiration, in fact a reduction in levels can also reduce the mitochondrial membrane potential. It is also involved in reactive oxygen species (ROS) production, in fact an increase in opening hemichannels can increase ROS production. This molecule interacts with other molecules, as the apoptosis induced factors, and this means that they also play a role in the integrity of mitochondria [89].

In asthma, increased mitochondrial dysfunction has been observed, and asthma specific cytokines (IL-4) are associated with increased mitochondrial damage [90]. Mitochondrial dysfunction has also been observed in COPD. Increasing Cx43 expression to stimulate the transfer of mitochondria producing power to damaged cells may become a new approach to treating lung disease symptoms. Thus, the transplantation of human induced pluripotent mesenchymal stem cells (hiPMSCs) to nicotine-intoxicated rats showed the transfer of mitochondria to airway epithelial cells in the setting of alveolar destruction and lung fibrosis [91].

## 11. Prospects for Therapeutic Interventions on Connexin Channels

One promising direction is the development of therapeutic approaches aimed at modulating the expression or activity of connexins to restore normal gap junction function. For example, interfering with the regulation of connexin-dependent pathways may help reduce inflammation and fibrosis in chronic obstructive pulmonary disease (COPD) or asthma. This can be achieved through the use of molecules that enhance or suppress the expression of certain types of connexins, or through the development of pharmacological agents capable of modulating their functional activity [92].

Connexins can also be considered as targets for gene therapy and genome editing to correct their dysfunction. Studies show that under certain conditions, the manipulation of connexin expression can lead to significant improvement in the condition of patients with lung diseases. Lung pathologies such as COPD, pulmonary fibrosis, and asthma are often associated with inflammatory processes and abnormal cellular communication. Recent studies show that the dysfunction of connexins may be important in the pathogenesis of these diseases. For example, connexins have been shown to play a role in the regulation of inflammatory and immune responses in lung tissue, as well as in healing and regeneration processes. One promising area of therapy is modulation of the expression and function of specific connexin subtypes in lung tissue. In particular, changing the expression level of Cx43 can have a significant impact on inflammatory responses and fibrogenesis processes [93].

Peptide mimetics are also considered in the context of the treatment of idiopathic pulmonary fibrosis. This condition is characterized by the uncontrolled growth of fibrous tissue, which limits the ability of the lungs to absorb oxygen. The main interest in peptide mimetics in the context of pulmonary pathologies is dictated by their ability to specifically affect the mechanisms of the disease, reducing inflammation, promoting tissue regeneration, and modulating the immune response. For example, in the treatment of COPD and asthma, they can be used to reduce inflammatory processes that play a key role in the progression of these diseases. The use of connexin inhibitors or mimetics to correct impaired communication in lung tissue offers a new therapeutic approach. For example, synthetic mimetics that imitate the functions of connexins can help improve intercellular communication and reduce inflammation. Specific connexin channel antagonists are also being developed that could be used to reduce pathological communication leading to disease [50,94].

In experimental models, the possibility of using Cx as endogenous negative regulators of autophagy is also being considered [95].

For example, the gap junction protein GJB3 is being considered as a therapeutic target for a wide range of cancer types, including lung adenocarcinoma where the knockdown of the protein inhibits the PI3K/AKT pathway and leads to a decrease in proliferation, migration, and viability of cancer cells [96].

## 12. Conclusions

Cx43 has been considered as a therapeutic target for multiples diseases, including lung cancer, skin disorders, corneal damage, ischemic heart injury, etc. However, inhibiting Cx43 function could be beneficial for preventing tumor cell growth or for improving tissue healing yet may have negative side effects due to its multifunctionality and direct impact on tissue homeostasis. It is also important to keep in mind that considering only one connexin as a target narrows the window of reality. For example, some lung development defects observed in mice with altered Cx43 expression can be restored with the help of induced expression of connexins 32 or 40.

Elucidating the role of intercellular gap junctions in the pathogenesis of respiratory diseases and a deeper understanding of molecular mechanisms underlying the activities of various connexins in gap junction channels will enable the prospective development of therapeutic approaches by either inhibiting or stimulating the activities of a certain connexin, while considering its critical functions in intercellular communications on the whole.
